# Anaerobic Corrosion of 304 Stainless Steel Caused by the *Pseudomonas aeruginosa* Biofilm

**DOI:** 10.3389/fmicb.2017.02335

**Published:** 2017-11-27

**Authors:** Ru Jia, Dongqing Yang, Dake Xu, Tingyue Gu

**Affiliations:** ^1^Department of Chemical and Biomolecular Engineering, Institute for Corrosion and Multiphase Technology, Ohio University, Athens, OH, United States; ^2^School of Materials Science and Engineering, Northeastern University, Shenyang, China

**Keywords:** *Pseudomonas aeruginosa*, biofilm, nitrate reducing bacterium, biocorrosion, 304 stainless steel, anaerobic corrosion

## Abstract

*Pseudomonas aeruginosa* is a ubiquitous bacterium capable of forming problematic biofilms in many environments. They cause biocorrosion of medical implants and industrial equipment and infrastructure. Aerobic corrosion of *P. aeruginosa* against stainless steels has been reported by some researchers while there is a lack of reports on anaerobic *P. aeruginosa* corrosion in the literature. In this work, the corrosion by a wild-type *P. aeruginosa* (strain PAO1) biofilm against 304 stainless steel (304 SS) was investigated under strictly anaerobic condition for up to 14 days. The anaerobic corrosion of 304 SS by *P. aeruginosa* was reported for the first time. Results showed that the average sessile cell counts on 304 SS coupons after 7- and 14-day incubations were 4.8 × 10^7^ and 6.2 × 10^7^ cells/cm^2^, respectively. Scanning electron microscopy and confocal laser scanning microscopy corroborated the sessile cell counts. The X-ray diffraction analysis identified the corrosion product as iron nitride, confirming that the corrosion was caused by the nitrate reducing biofilm. The largest pit depths on 304 SS surfaces after the 7- and 14-day incubations with *P. aeruginosa* were 3.9 and 7.4 μm, respectively. Electrochemical tests corroborated the pitting data.

## Introduction

Microbiologically influenced corrosion (MIC), also known as biocorrosion, is initiated and accelerated by microbes (Coetser and Cloete, 2005; Krishnamurthy et al., 2015). Biofilms were responsible for MIC (Li Y. et al., 2016; Qi et al., 2016; Wu et al., 2016; Xu et al., 2017a). They are communities of microbes embedded in extracellular polymeric substances that protect sessile cells from the outside environments (Stewart and Costerton, 2001; Hall-Stoodley et al., 2004; Ghanbari et al., 2016; Kirchhoff et al., 2017). More than 20% of metal corrosion damages was attributed to MIC (Flemming, 1996). MIC is a serious problem in many settings, such as medical implants (Jia et al., 2017g), the oil and gas industry (Liu et al., 2016), marine environments (Ramírez et al., 2016), and water utilities (Jia et al., 2017a). Stainless steel is a commonly used metal in many industries due to their corrosion resistance (Sedriks, 1996). Stainless steel is also a biocompatible metal which is used in orthopedic and dental implants because this corrosion resistant metal with high strength is biocompatible (Manam et al., 2017). Stainless steel is not immune from MIC pitting attacks, albeit to a much less degree compared with carbon steel (Li L.M. et al., 2016; Manam et al., 2017). Stainless steels such as types 304 and 316 have no antibacterial properties and therefore, biofilms may cause corrosion when the stainless steel passivation film is damaged (Lopes et al., 2006). It has been reported that the corrosion products of stainless steels in the human body fluid medium were harmful to the human body (Manivasagam et al., 2010).

*Pseudomonas* species are ubiquitous in nature. They are also present in some medical settings (San et al., 2014; Sanchez et al., 2014). *Pseudomonas aeruginosa* is a Gram-negative facultative bacterium. Persistent biofilms are found on stainless steel in drinking water systems (Moritz et al., 2010). *P. aeruginosa* biofilms can also form on catheters, contact lenses, and cystic fibrosis (CF) infected lungs (Pusic et al., 2016). A number of studies have showed aerobic corrosion of *P. aeruginosa* on stainless steels (Morales et al., 1993; Lou et al., 2016; Xu et al., 2017b). However, there is a lack of anaerobic *P. aeruginosa* corrosion studies in the literature. *P. aeruginosa* is a facultative bacterium. In an open-to-air system, the top layer of a *P. aeruginosa* biofilm is aerobic, but the bottom layer may be anaerobic. *P. aeruginosa* biofilms grow in CF lungs are anaerobic (Yoon et al., 2002). They usually infect medical implants anaerobically (Widmer, 2001). In anaerobic respiration, *P. aeruginosa* uses nitrate or nitrite as a terminal electron acceptor (Yoon et al., 2002).

The present work was aimed at studying the MIC by a wild-type *P. aeruginosa* (PAO1) biofilm cultured as a nitrate reducing bacterium (NRB) biofilm on 304 stainless steel (304 SS) in a strictly anaerobic environment. The biofilm and surface morphologies were examined under scanning electron microscopy (SEM), confocal laser scanning microscopy (CLSM), and infinite focus microscopy (IFM). Linear polarization resistance (LPR) and electrochemical impedance spectroscopy (EIS) were utilized to investigate the corrosion behaviors of 304 SS submerged in a nitrate containing culture medium with and without *P. aeruginosa*. The corrosion products were analyzed using X-ray diffraction (XRD).

## Materials and Methods

### Bacterium, Culture Medium, Coupon, Chemicals, and MIC Testing

The wild-type *P. aeruginosa* (strain PAO1) was used. The seed culture was grown in the Luria-Bertani medium supplemented with KNO_3_ (LB-NO_3_ medium). The LB-NO_3_ medium was consisted of 10 g tryptone, 5 g yeast extract, 5 g NaCl, and 10 g KNO_3_ in 1 L deionized water. The culture medium pH was adjusted to 7.0 by adding a NaOH solution. One hundred ppm (w/w) L-cysteine was added to the culture medium as an O_2_ scavenger to mitigate any possible O_2_ ingress. The culture medium and vials were autoclaved at 121°C for 20 min. The L-cysteine solution was filter sterilized using a 0.22 μm Stericup filter (Millipore, Bedford, MA, United States). All liquid solutions were sparged with filter-sterilized N_2_ to remove dissolved oxygen for at least 1 h. Disk-shaped coupons used in this work were cut from a 304 SS rod purchased from McMaster-Carr (Aurora, OH, United States). The 1 cm^2^ top surface was exposed while all other surfaces were protected by polytetrafluoroethylene paint, which is chemically inert and corrosion proof (Zhao et al., 2004). The composition of 304 SS was (% by mass): C 0 – 0.08, Cr 17.5 – 24.0, Co 0 – 0.3, Cu 0 – 1.0, Mn 0 – 2.0, Mo 0 – 2.5, Ni 8.0 – 15.0, N 0 – 0.1, P 0 – 0.2, S 0 – 0.3, Si 0 – 1.0, and Fe balance.

Coupons were abraded with a series (180, 400, and 600 grit) of abrasive papers. They were then washed with 100% isopropanol and dried with N_2_ gas under UV light for at least 20 min. A N_2_-filled chamber provided an anaerobic environment for all anaerobic manipulations. Chemicals were purchased from either Fisher Scientific (Pittsburgh, PA, United States) or Sigma-Aldrich (St. Louis, MO, United States). Anaerobic MIC testing was conducted in 125 ml anaerobic vials (Wheaton Industries, Inc., Millville, NJ, United States). In each 125 ml anaerobic vial, 100 ml LB-NO_3_ medium with and without 2 ml anaerobic *P. aeruginosa* seed culture, and four replicate coupons were added. The initial planktonic cell concentration introduced by the seek culture during inoculation was 10^5^–10^6^ cells/ml. Vials were then sealed and incubated at 37°C. After 7 and 14 days, coupons were taken out for biofilm and corrosion analyses.

### Sessile Cell Count and Biofilm Observation

The sessile cell count on the coupon surface was enumerated with a hemocytometer under a 400X optical microscope (Bjornson and Michael, 1971). Coupons were slightly rinsed in a pH 7.4 phosphate buffered saline (PBS) buffer solution to remove planktonic cells and the culture medium. The biofilm from a coupon was transferred to a test tube using a small sterilized brush. The cells were suspended in a 10 ml pH 7.4 PBS solution. After that, the applicator and the coupon were also put into the 10 ml PBS solution. The 10 ml PBS solution, the coupon and the applicator were placed in into a 50 ml test tube and vortexed for 0.5 min to distribute cells evenly in the solution before counting. The biofilm morphology on coupon surfaces was examined under SEM (Model JSM-6390 SEM, JEOL, Tokyo, Japan) and its energy-dispersive X-ray spectroscopy (EDS) accessary (Jia et al., 2017e). CLSM (Model LSM 510 microscopy, Carl Zeiss, Jena, Germany) was used to observe live and dead cells in biofilms and biofilm thickness (Jia et al., 2017a). The corrosion product layer was analyzed by XRD analysis performed on a Discover D8 machine with a Co K-alpha X-ray tube (Bruker, Karlsruhe, Germany).

### Pitting Observation

The biofilms and corrosion products on a coupon was removed using a fresh Clarke’s solution according to ASTM G1-03 (Jia et al., 2017f). After that, coupons were rinsed with deionized water, 100% isopropanol and then dried with N_2_ gas for pit observation. The pit morphology was examined under SEM. The maximum pit depth was measured under IFM (Model ALC13, Alicona Imaging GmbH, Graz, Austria).

### Electrochemical Measurements

Linear polarization resistance and EIS tests were conducted in 450 ml glass cells equipped with a VersaSTAT 3 potentiostat (Princeton Applied Research, Oak Ridge, TN, United States). The glass cells each contained 350 ml culture medium with and without *P. aeruginosa* inoculation. A platinum mesh served as the counter electrode. A saturated calomel electrode (SCE) acted as the reference electrode. For LPR measurements, the potential was scanned at a rate of 0.167 mV/s from -10 to +10 mV vs. the open circuit potential (OCP). EIS was scanned with a 10 mV sinusoidal voltage in a frequency range of 10^-2^ to 10^5^ Hz at a stable OCP. EIS results were analyzed using the ZSimDemo 3.30d (EChem Software, Ann Arbor, MI, United States).

## Results

### Sessile Cell Count and Biofilm Observation

**FIGURE 1:**
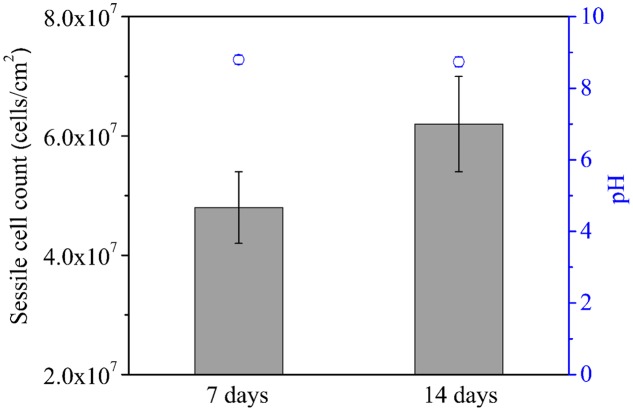
Sessile cell counts (bars) on the coupon surface and pH values (circles) of the culture medium after 7 and 14 days of incubation. Scatter bands are standard deviations of 4 independent samples.

**FIGURE 2:**
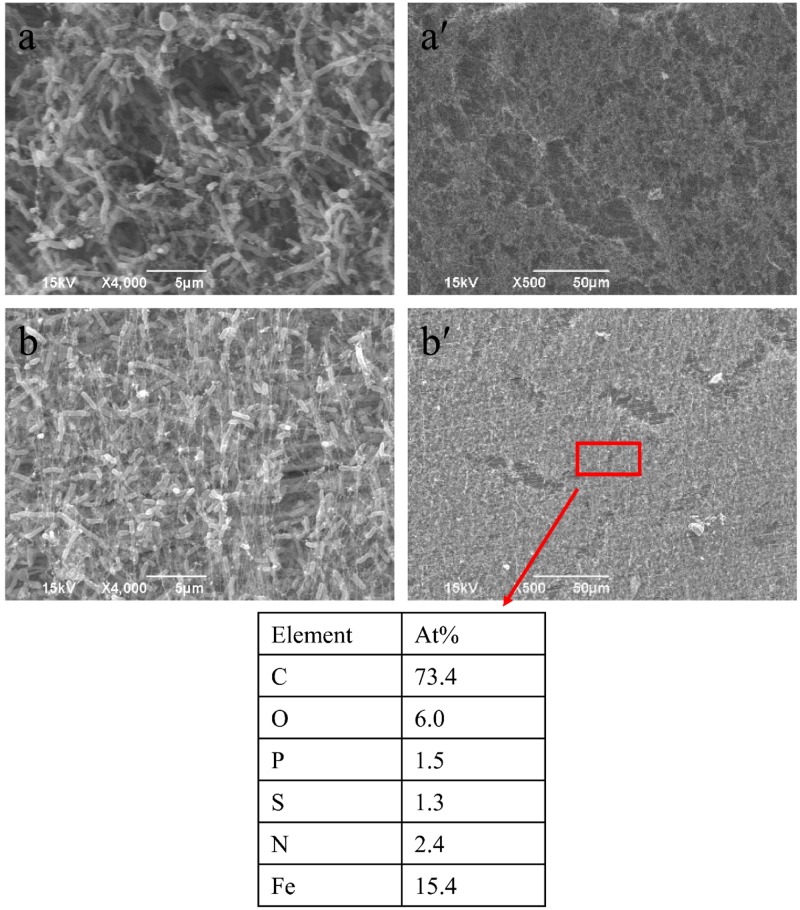
. Biofilm SEM images of 304 SS exposed to *Pseudomonas aeruginosa* for 7 days **(a,a′)** and 14 days **(b,b′)**.

**FIGURE 3:**
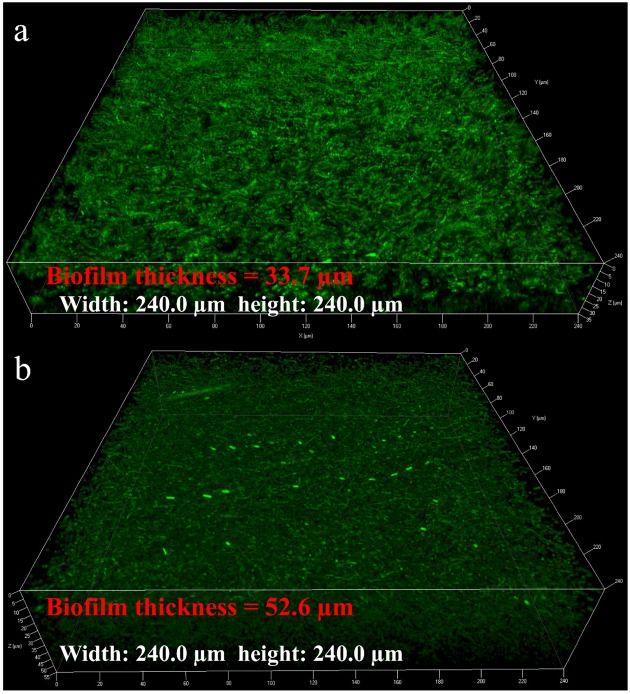
Confocal laser scanning microscopy (CLSM) images of the coupon surface after incubation for 7 days **(a)** and 14 days **(b)**.

The average sessile cell counts on the 304 SS coupons after the 7- and 14-day incubations were 4.8 × 10^7^ and 6.2 × 10^7^ cells/cm^2^, respectively (**Figure 1**). These large numbers suggest that the NRB biofilm grew well on 304 SS. **Figure 1** shows that the pH values in the bulk liquids after the 7- and 14-day incubations. Both pH values were above 8.5. With this kind of high pH, acid attack effect was not a factor in the corrosion. The pH value in the abiotic medium after the 14-day incubation remained at the initial pH 7.0. Biofilm SEM images in **Figure 2** show the biofilm morphology on the stainless steel coupon surfaces. Sessile cells embedded in biofilms can be seen on the surface of 304 SS. Abundant rod-shaped *P. aeruginosa* sessile cells were observed after the 7- and 14-day incubation tests. The density of sessile cells after 14 days (**Figure 2b′**) was higher than that after 7 days of incubation (**Figure 2a′**). The EDS analysis (indicated in red) of the 14-day incubation coupon revealed that the biofilm and corrosion products were composed of carbon, oxygen, phosphate, sulfur, nitrogen, and iron elements. CLSM was also used to observe sessile cells and biofilm thickness on coupons. **Figure 3** shows that live cells (green dots) were very dense on the coupons. The biofilm was thicker after the 14-day incubation than that after the 7-day incubation. The results supported the sessile cell counts in **Figure 1**.

### Corrosion Analyses after the Incubation Test

**FIGURE 4:**
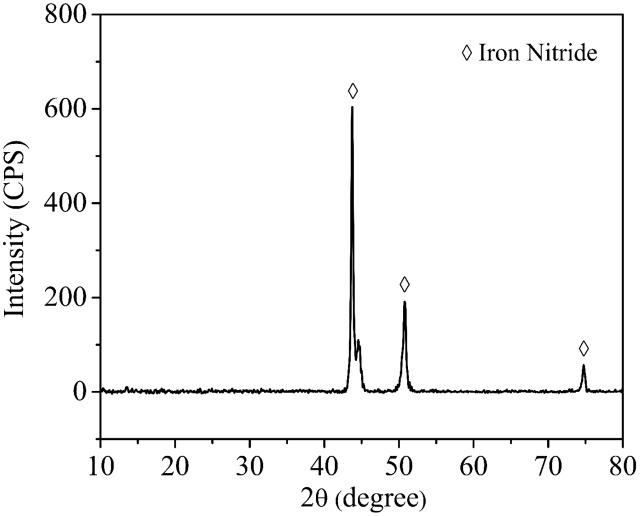
X-ray diffraction (XRD) patterns of corrosion products on 304 SS surface after the 14-day incubation.

**FIGURE 5:**
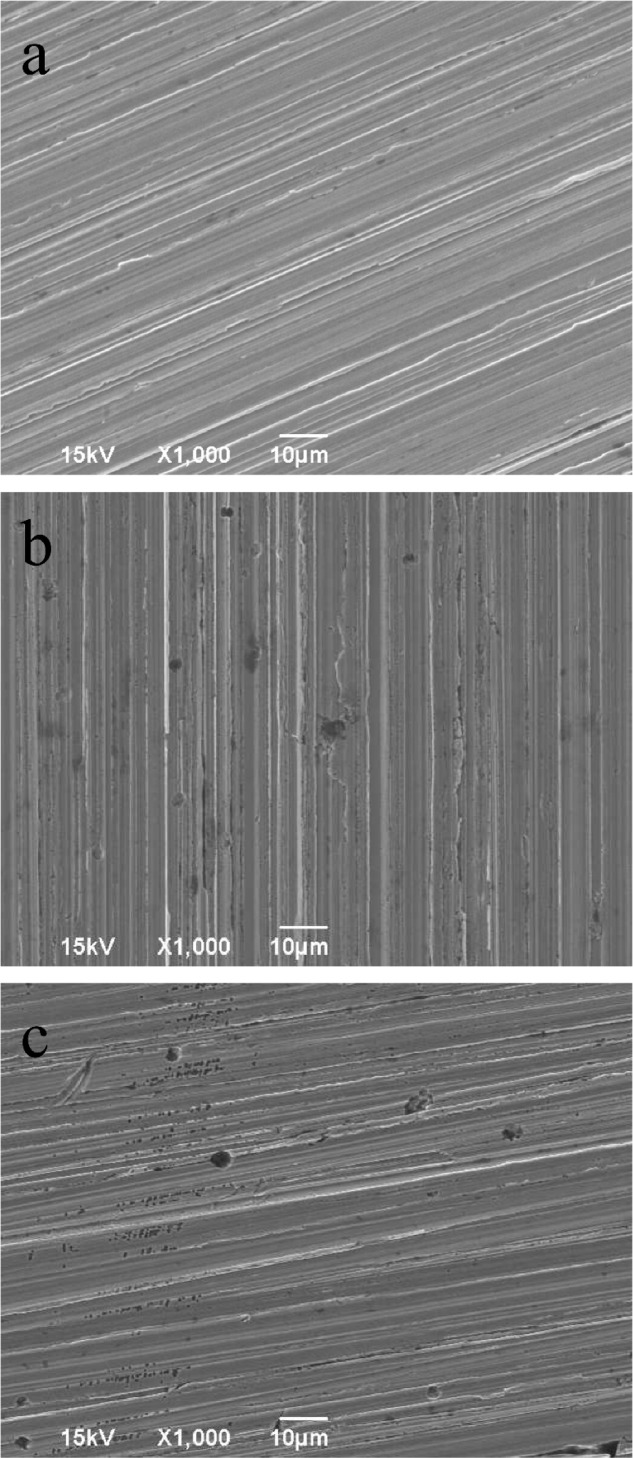
Pit images: **(a)** abiotic control incubated for 14 days, **(b)** inoculated coupon incubated for 7 days, and **(c)** inoculated coupon incubated for 14 days.

**FIGURE 6:**
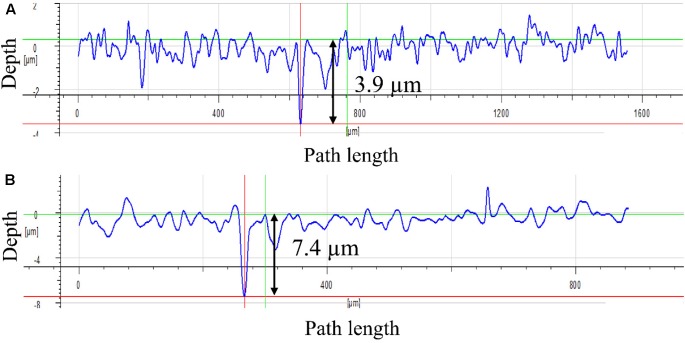
Maximum pit depth on the coupon surface under IFM: **(A)** after 7-day incubation and **(B)** after 14-day incubation.

**Figure 4** shows the corrosion product on a 14-day coupon incubated with *P. aeruginosa*. XRD revealed that iron nitride was the only corrosion product on the coupon surface. After the coupon was cleaned using Clarke’s solution, the pit morphology was observed under SEM. **Figure 5a** shows the coupon surface from the abiotic medium. No apparent corrosion pits were found in the image that showed parallel polishing lines. In the inoculated medium, some small pits can be seen on the coupon surface after the 7-day incubation in **Figure 5b**. More severe pitting corrosion is seen on the coupon incubated for 14 days (**Figure 5c**) compared with that for 7 days (**Figure 5b**). The maximum pit depth of the coupons incubated in the *P. aeruginosa* inoculated medium was examined under IFM (**Figure 6**). The abiotic control coupon did not exhibit any pitting (image not shown). The largest pit depth on 304 SS surface in the inoculated media after the 7- and 14-day incubations were 3.9 and 7.4 μm, respectively.

### Electrochemical Tests during the Incubation Test

**FIGURE 7:**
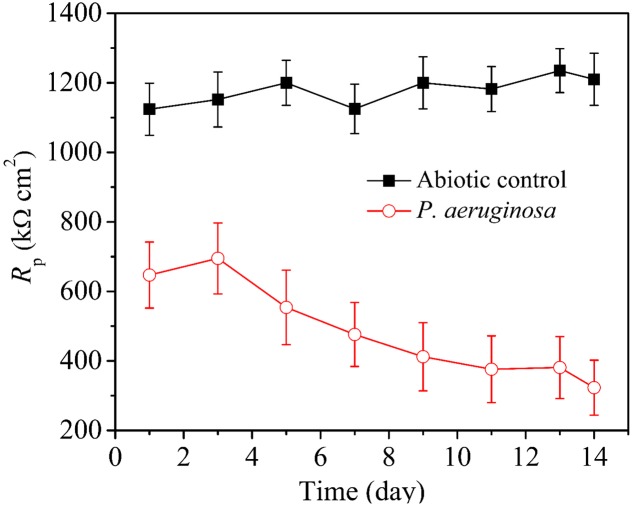
Variations of LPR polarization resistance under different conditions vs. time during the 14-day incubation with and without *P. aeruginosa*. Scatter bands are standard deviations of 3 independent samples.

Pitting corrosion testing requires several days of corrosion before the pit depth becomes significant for measurements. However, electrochemical tests do not need this lead time. LPR is an efficient, non-destructive and rapid method that can be used to determine corrosion rates (Zou et al., 2011). **Figure 7** shows variations of LPR polarization resistance (*R*_p_) under the abiotic and inoculated media vs. time during the 14-day incubation. A larger *R*_p_ value means higher corrosion resistance. The *R*_p_ values of 304 SS were quite high in the abiotic medium and varied very little during the 14-day incubation. This was expected because there was no corrosion going on. However, in the presence of *P. aeruginosa*, the *R*_p_ values were lower, indicating corrosion by *P. aeruginosa*. The *R*_p_ values in the inoculated medium increased during the first 3 days of incubation and then gradually decreased from the third day during the 14-day incubation.

**FIGURE 8:**
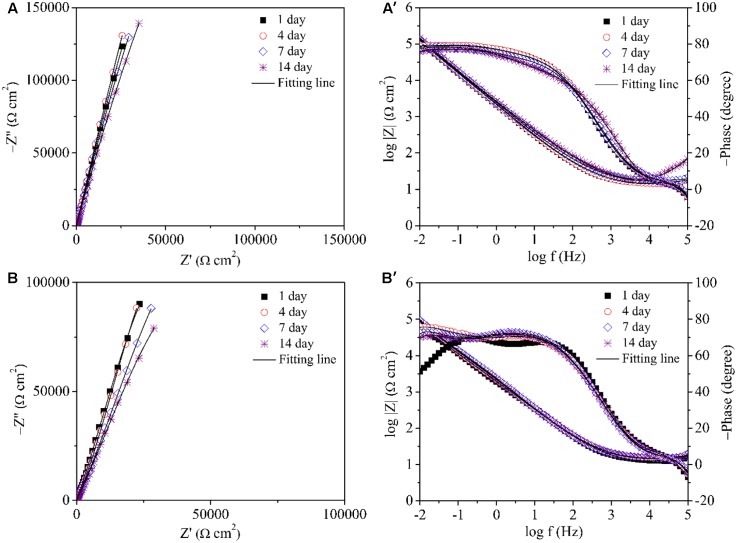
Nyquist and Bode plots for the coupons during the 14 days of incubation with and without *P. aeruginosa*: **(A,A′)** abiotic control and **(B,B′)** inoculated with *P. aeruginosa*.

Electrochemical impedance spectroscopy is another non-destructive electrochemical test that can be adopted to study electrochemical reactions in MIC on a coupon (Manohar et al., 2008). The impedance spectra of 304 SS in the abiotic medium are shown in **Figures 8A,A′**. The Nyquist plot in **Figure 8A** shows similar impedance loops with incubation time. Only one time constant exhibited in **Figure 8A′**. The impedance spectra of 304 SS in the *P. aeruginosa* inoculated medium are shown in **Figures 8B,B′**. The diameters of the impedance loops in the Nyquist plots **Figure 8B** decreased with the incubation time, indicating increased corrosion. In the phase plots (**Figure 8B′**), two peaks showed up. **Table 1** lists the fitted EIS parameters of coupons obtained on four different days during 14 days of incubation. *R*_s_ is the solution resistance. *R*_b_ stands for the biofilm or corrosion product film resistance. *R*_ct_ represents the charge transfer resistance. *Y* and *n* are constant phase element parameters. The EIS results for the coupons immersed in the abiotic medium were fitted using the R(QR) circuit, whereas those for the coupons immersed in the *P. aeruginosa* inoculated medium were fitted with the R(Q(R(QR))) equivalent circuit (Xu et al., 2017b). The *P. aeruginosa* broth had a decreased *R*_ct_ compared with the abiotic medium, indicating corrosion acceleration by *P. aeruginosa*. Continued incubation in the inoculated medium resulted in a gradual decrease in *R*_ct_ (i.e., increase corrosion) during the 14-day incubation.

**Table 1:** Electrochemical impedance spectroscopy (EIS) parameters of coupons obtained on four different days during the 14 days of incubation (standard deviations from 3 independent samples).

Day	*R*_s_ (Ω cm^2^)	*Y*_b_ (Ω*^-^*^1^ cm*^-^*^2^ s^n^)	*n*_b_	*R*_b_ (Ω cm^2^)	*Y*_dl_ (Ω*^-^*^1^ cm*^-^*^2^ s^n^)	*n*_dl_	*R*_ct_ (MΩ cm^2^)
**304 SS in the abiotic medium**							
1	16.1 ± 2.3				0.0009 ± 0.0003	0.85 ± 0.07	1.1 ± 0.3
4	15.8 ± 2.1				0.0008 ± 0.0002	0.87 ± 0.14	1.2 ± 0.2
7	17.5 ± 2.5				0.0008 ± 0.0003	0.81 ± 0.19	1.1 ± 0.3
14	17.4 ± 2.8				0.0006 ± 0.0002	0.84 ± 0.12	1.1 ± 0.2
**304 SS in the PA inoculated medium**							
1	14.6 ± 2.2	0.0008 ± 0.0004	0.79 ± 0.08	17.1 ± 1.8	0.0012 ± 0.0006	0.91 ± 0.05	0.5 ± 0.2
4	15.5 ± 1.9	0.0009 ± 0.0005	0.77 ± 0.09	17.9 ± 2.2	0.0013 ± 0.0007	0.78 ± 0.11	0.5 ± 0.2
7	15.1 ± 1.6	0.0006 ± 0.0003	0.89 ± 0.13	21.6 ± 1.7	0.0015 ± 0.0009	0.76 ± 0.09	0.4 ± 0.1
14	15.7 ± 1.7	0.0005 ± 0.0002	0.79 ± 0.07	29.6 ± 2.1	0.0011 ± 0.0008	0.81 ± 0.08	0.3 ± 0.1


## Discussion

Biofilms are directly responsible for MIC due to their metabolic activities or secreted metabolites. Generally, there are two main types of anaerobic MIC (Gu, 2014). In the first type of MIC, exogenous non-oxygen oxidants such as nitrate, sulfate, CO_2_, etc. serve as the terminal electron acceptor. Sessile cells underneath a biofilm have less access to the organic carbon molecules in the bulk liquid due to diffusional resistance of the biofilm and consumption by top-layer sessile cells. Therefore, those sessile cells near the bottom are forced to switch to elemental iron as the electron donor (Xu and Gu, 2014). Because elemental iron is insoluble, bacterial biofilms have to transfer electrons from extracellular elemental iron oxidation across the cell wall to the bacterial cytoplasm for the reduction of an oxidant under biocatalysis (Xu et al., 2013). A previous study showed that *P. aeruginosa* biofilms corroded carbon steel more aggressively when the culture medium had less organic carbon despite the fact that the organic carbon shortage led to a decreased sessile cell count. This was because *P. aeruginosa* sessile cells switched from organic molecules to Fe^0^ as the electron donor for respiration, which means these cells became corrosive (Jia et al., 2017h). Fe^0^ used as an electron donor for microbial growth was already proven in evolutionary microbiology (Biswas and Purnendu, 2005). Planktonic cells are surrounded by a body of water, thus, they cannot transport extracellular electrons released by a steel surface due to iron oxidation (Jia et al., 2017c). This means planktonic cells cannot directly cause this type of MIC, which can be called extracellular electron transfer MIC (EET-MIC) (Jia et al., 2017b).

The second type of MIC is caused by corrosive metabolites secreted by microbes such as organic acids and proton (Gu, 2012). The reduction of these oxidants (e.g., proton reduction) is also electrochemical. This type of MIC can be called metabolite MIC (M-MIC) (Jia et al., 2017b). The local pH underneath a biofilm can be much more acidic than that in the bulk culture medium due to the local secretion of organic acids. Protons can attack iron extracellularly without biocatalysis. In this work, the pH measured in the inoculated medium after the 14-day incubation were all above 8.5. Although the pH underneath biofilms can differ from the bulk by as much as two units (Hidalgo et al., 2009), the relative high pH in the bulk suggested that acid attack was unlikely a factor. Therefore, the corrosion of 304 SS was due to *P. aeruginosa* respiration using the electrons released by the elemental iron oxidation for nitrate reduction similar to carbon steel corrosion by nitrate reducing *Bacillus licheniformis* (Xu et al., 2013). Iron was oxidized as shown in Reaction 1. Biological denitrification can reduce nitrate to nitrogen gas (Reaction 2) (Ghafari et al., 2008; Jia et al., 2017h):

$$Fe\to {Fe}^{2+}+{2e}^{-}$$

$$2{{NO}_{3}}^{-}+{10e}^{-}+{12H}^{+}\to N_{2}+{6H}_{2}O$$

It was found that N_2_ can be reduced to nitride to form iron nitride in the presence of K^+^ cation as a catalyst in the culture medium (Rodriguez et al., 2011). The XRD result in this work also supported this. The anaerobic *P. aeruginosa* biofilm caused significant pitting corrosion of 304 SS. It was found that the pit depth of 304 SS caused by *P. aeruginosa* biofilms after 14 days of incubation under an aerobic condition was around 0.2 μm (Yuan and Pehkonen, 2007). In comparison, the maximum pit depth on 304 SS coupon surface in the anaerobic *P. aeruginosa* corrosion was 7.4 μm after the 14-day incubation which was much larger. The more severe pitting corrosion under the anaerobic condition could be due to biofilm’s weakening of the passivation film (Lopes et al., 2006) that could not be repaired in the absence of O_2_. 304 SS anaerobic corrosion caused by sulfate reducing bacteria (SRB) have also been reported in the literatures. SRB are considered culprits for MIC in many industries (Jia et al., 2017d). It was found that *Desulfovibrio vulgaris* led to a maximum pit depth of 4.8 μm after a 7-day incubation (Zhang et al., 2015) compared with 3.9 μm caused by *P. aeruginosa* in this work. Zhang et al. (2012) studied anaerobic MIC of 304 SS using a field SRB biofilm. Based on corrosion resistance from EIS, their SRB corrosion rate was several times higher than that in this work.

## Conclusion

The experimental results in this work showed that nitrate reducing *P. aeruginosa* formed robust biofilms on 304 SS coupons in the anaerobic condition. The 304 SS anaerobic corrosion by *P. aeruginosa* was reported for the first time. Significant MIC pitting corrosion was found on the coupon surface. LPR and EIS measurements confirmed the anaerobic corrosion caused by *P. aeruginosa* on 304 SS.

## Author Contributions

DX and TG conceived and designed the experiments. RJ and DY performed the experiments. RJ analyzed the data. RJ, DY, DX, and TG wrote the paper. All authors participated in the discussion about the results and the manuscript.

## Conflict of Interest Statement

The authors declare that the research was conducted in the absence of any commercial or financial relationships that could be construed as a potential conflict of interest.
